# Supplementary material for: Early warning model construction and validation for urinary tract infection in patients with neurogenic lower urinary tract dysfunction (NLUTD): a retrospective study
Source: PeerJ. 2022 May 5;10:e13388. doi: 10.7717/peerj.13388 (PMC9080428; doi:10.7717/peerj.13388)
Supplement: Supplemental Information 2 [file peerj-10-13388-s002.docx]

**Supplementary material Codebook of categorical data for all variables in the raw data**

| Variable | Assignment method |
| --- | --- |
| Independent variable |  |
| Combined with hypertension | Yes =1 , No =0 |
| Combined with diabetes | Yes =1 , No =0 |
| Combined with urinary calculi | Yes =1 , No =0 |
| Neurogenic bladder type | Detrusor overactivity=1, Detrusor inactiviety type=2, Mixed type=3 |
| Catheterization method | Indwelling catheterization =1 , intermittent catheterization/voiding =0 |
| Preventive use of antibacterial drug | Yes =1 , No =0 |
| Bladder training intervention time | ≥2 weeks =1 , <2 weeks =0 |
| Hypoproteinemia | Yes =1 , No =0 |
